# Scheuermann's Disease in Young Adults: A Case Report

**DOI:** 10.7759/cureus.31803

**Published:** 2022-11-22

**Authors:** Arpit Jain, Amit Saoji

**Affiliations:** 1 Department of Orthopaedics, Jawaharlal Nehru Medical College, Datta Meghe Institute of Medical Sciences, Wardha, IND; 2 Department of Orthopaedic Surgery, Jawaharlal Nehru Medical College, Datta Meghe Institute of Medical Sciences, Wardha, IND

**Keywords:** scheuermann's disease, lumbar, end plate, kyphosis, scoliosis, posture

## Abstract

Scheuermann kyphosis, also known as Scheuermann disease, juvenile kyphosis, or juvenile discogenic disease, is a condition involving an abnormal, excessive curvature of the spine. It involves both the vertebral bodies and discs of the spine and is characterized by anterior wedging of greater than or equal to 5 degrees in three or more adjacent vertebral bodies. Type 1 Scheuermann's disease involves the thoracic spine, whereas type 2 involves both the thoracic and lumbar spine. Although no definitive cause for Scheuermann’s disease has been found, we have reported a case that may explain further about this disease. This article elucidates a case of a 19-year-old boy experiencing pain in the lower back and showing various signs and symptoms of Scheuermann’s disease and the diagnosis and steps taken by doctors toward its treatment.

## Introduction

Scheuermann's disease is a vertebral disorder that affects the anterior wedging of the spine. The hunchback appearance is a common way to identify the illness. Trauma, developmental abnormalities, degenerative disc disease, inflammatory diseases, viral diseases, and iatrogenic factors can all contribute to kyphosis development.

It is named after Dr. Hans-Gerd von Scheuermann, who first described the disease in 1961. The disease can also affect the arms, hands, and fingers, as well as the hip and knee joints. There are several different types of Scheuermann's disease, including epiphyseal and atrophic. Most patients with Scheuermann's disease have no signs or symptoms when they have mild cases of the disease.

Scheuermann's disease presents a wide range of the clinical spectrum, the most common being the stunted growth of the endplate, lower back pain, and osteoporosis. Alternations in disc shape are a frequent sight. Radiographic results usually indicate anterior wedging of vertebral bodies, irregularity of endplate, and disc alternations due to the gradual weakening of cartilage by genetic background. The term "juvenile kyphosis" is another name for Scheuermann's disease [1]. The kyphotic deformity is mainly caused by poor posture, negligence in posture correction, and delay in diagnosis and treatment. Radiographic results usually indicate anterior wedging of vertebral bodies, irregularity of endplate, and disc alternations due to the gradual weakening of cartilage by genetic background [2]. Majorly thoracic region is affected, but the lumbar can also be the affected region. Genetic anomalies may be linked to Scheuermann's disease, even though its etiology is unknown. There are chances of Scheuermann's disease being more prevalent in monozygotic twins than in dizygotic twins [1]. Between 0.4% and 10% of the population are thought to be affected by this condition [3]

Scheuermann's disease is often noticed in teenagers from ages 13 to 18. The primary treatment comprises conservative methods, such as supplementations, medicines, physiotherapy, and posture correction. Brace therapy proves to be successful if the diagnosis is made early [4]. Surgery is usually not suggested in early diagnosis and is strictly limited when conservative methods fail. A Stagnara angle of more than 70° or 75° requires surgical intervention when there are neurologic impairments, curve advancement despite conservative therapy, and worsening pain sensations [5]. Scheuermann's disease can be divided into Type 1 and Type 2. Type 1 Scheuermann's disease revolves around pain or problems in the thoracic spine. In contrast, type 2 Scheuermann's disease involves thoracic and lumbar spine pain [1].

## Case presentation

A 19-year-old man came in complaining of lower back pain, numbness of lower limbs, radiating pain to the legs for one week, and on-and-off episodes of excruciating back pain. Pain is progressive and increases on movement. In both lower limbs, tingling sensations occur in conjunction with pain. The patient had back pain after continuous long sitting hours and strenuous exercise. The patient, as mentioned earlier, had no recent history of trauma, an accident while heavy weightlifting, a fall from height, or any birth anomaly.

On General examination, the patient was examined in a supine position with both ASIS (anterior superior iliac spine) at the same level, where he was conscious and well-oriented the whole time. On inspection LS's (lumbosacral) spinal curvature was normal, and no scars or sinuses were seen. Active toe movement and distal circulation were normal. The patient had a regular pulse of 88/minutes, respiratory rate of 15, and blood pressure of 120/80 mm of Hg. The patient had no history of loss of consciousness, vomiting, or internal bleeding. A thorough physical examination reveals that the patient has paraspinal muscle spasms over the lumbar region, with no signs of hypoesthesia. On doing the straight leg raise test, it was observed that the patient could lift his left leg to the extent of 50° and right leg to the extent of 60°. No symptoms of distal neurovascular deficit were seen.

Clinical examination revealed that the erythrocyte sedimentation rate (ESR) value was 12, which was normal (0 to 22 mm/hr). The calcium was 8.2, slightly less than the normal count (8.6 to 10.3 mg/dl). Creatinine was normal but by a slight margin, i.e., 0.7 (0.74 to 1.35mg/dl). Sodium was 139, which is also normal (139 to 145 milliequivalents/liter). Potassium was 3.7, normal (3.6 to 5.2 millimoles/liter). Table 1 shows the blood sample in a tabular form for better understanding. 

**Table 1:** Patient's blood report sample Hb: Hemoglobin, MCHC: Mean corpuscular hemoglobin concentration, MCV: Mean corpuscular volume, MCH: Mean corpuscular hemoglobin, RBC: Red blood cells, WBC: White blood cells, HCT: Hematocrit test, ESR: Erythrocyte sedimentation rate, IPTH: Parathyroid hormone

Blood Tests	Required Value	Obtained Value	Analysis
Hb	13 to 17	14.6	Normal
MCHC	32 to 36	34.8	Normal
MCV	80 to 100	84.9	Normal
MCH	27 to 31	29.5	Normal
RBC Count	4.7 to 6.1	4.93	Normal
WBC Count	4,500 to 11,000	6000	Normal
Platelet Count	1.50 to 4.50	2.14	Normal
HCT	38.3 to 48.6	41.9	Normal
ESR	0 to 22	12	Normal
Calcium	8.6 to 10.3	8.2	Abnormal
IPTH	10 to 55	49.8	Normal
Urea	6 to 24	14	Normal
Creatinine	0.7 to 1.3	0.7	Normal
Sodium	139 and 145	139	Normal
Potassium	3.6 to 5.2	3.7	Normal
Alkaline Phosphatase	30 to 120	106	Normal
ALT	7 to 56	32	Normal
AST	8 to 33	33	Normal
Total Protein	6.0 to 8.3	7.3	Normal
Albumin	3.4 to 5.4	4.8	Normal
Total Bilirubin	0.1 to 1.2	0.7	Normal
Vitamin B12	160 to 950	428	Normal
Vitamin D	20 and 40	34.5	Normal

**Figure 1:**
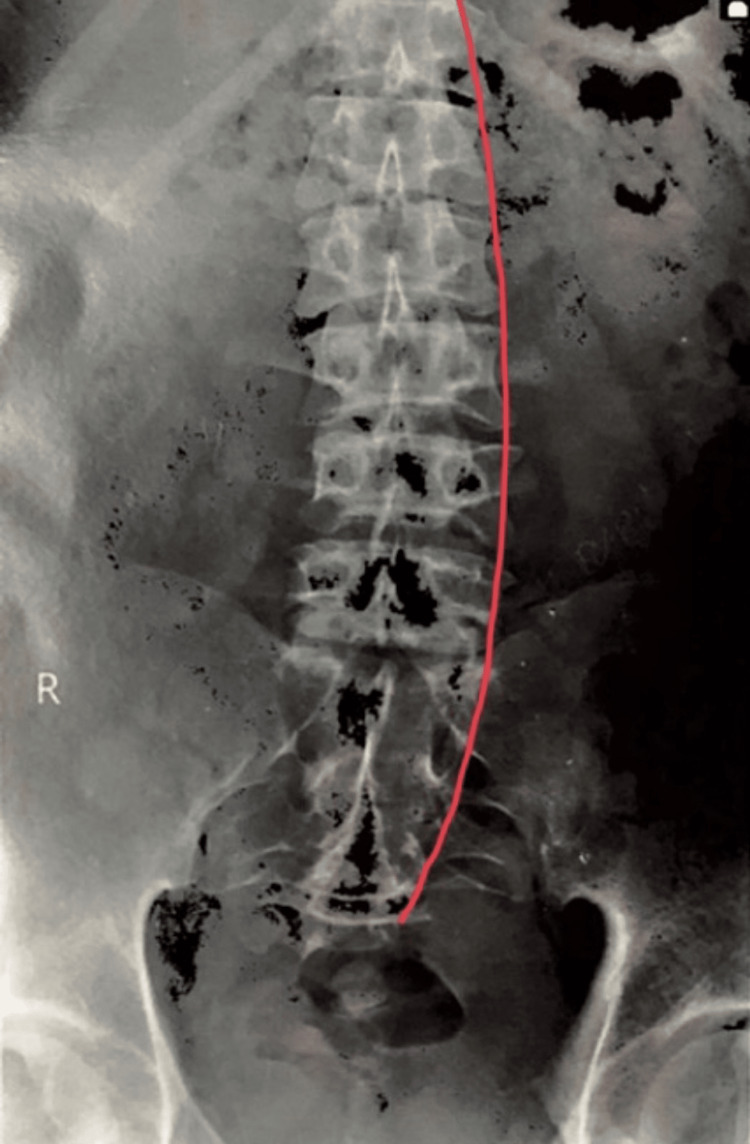
X-Ray shows a slight bent in the vertebra The red line shows a curve in the spine Image Credit: Author: Arpit Jain

**Figure 2:**
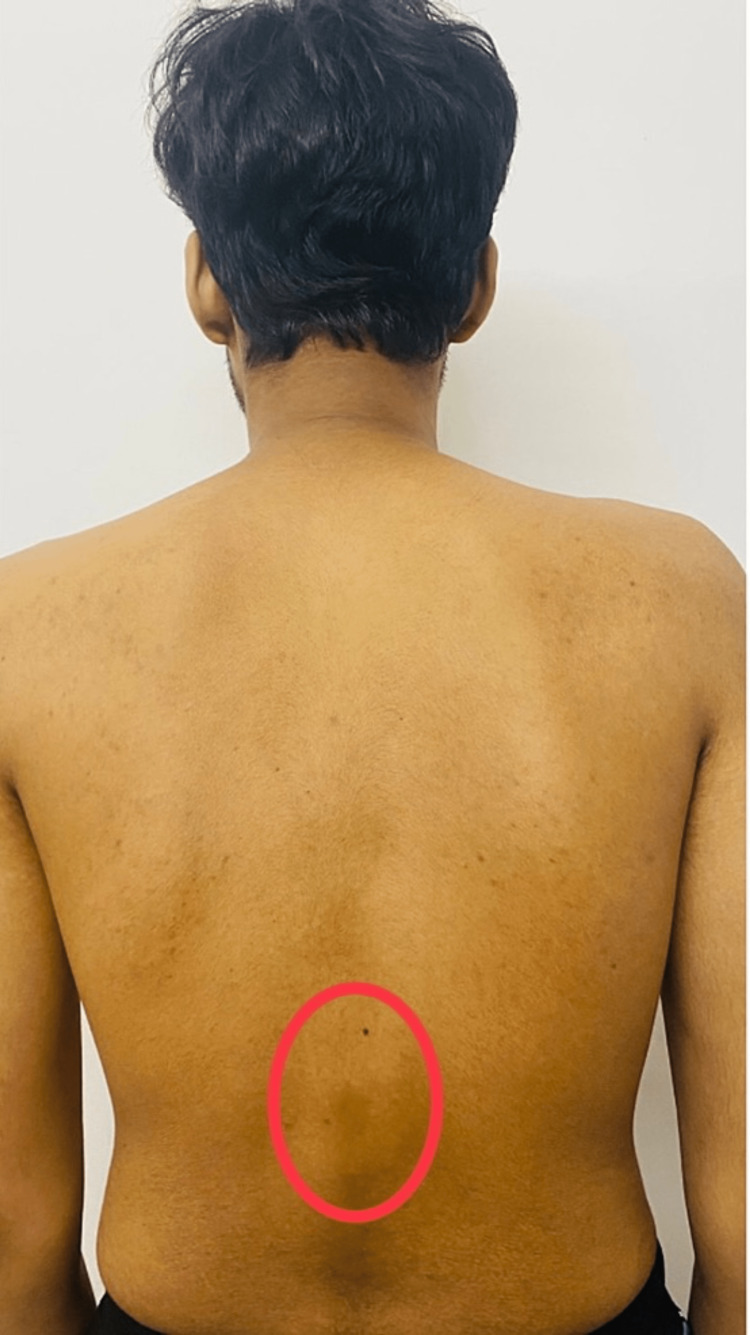
The image showcases the kyphotic deformity in the back The red circle shows kyphotic deformity Image Credit: Author: Arpit Jain

**Figure 3:**
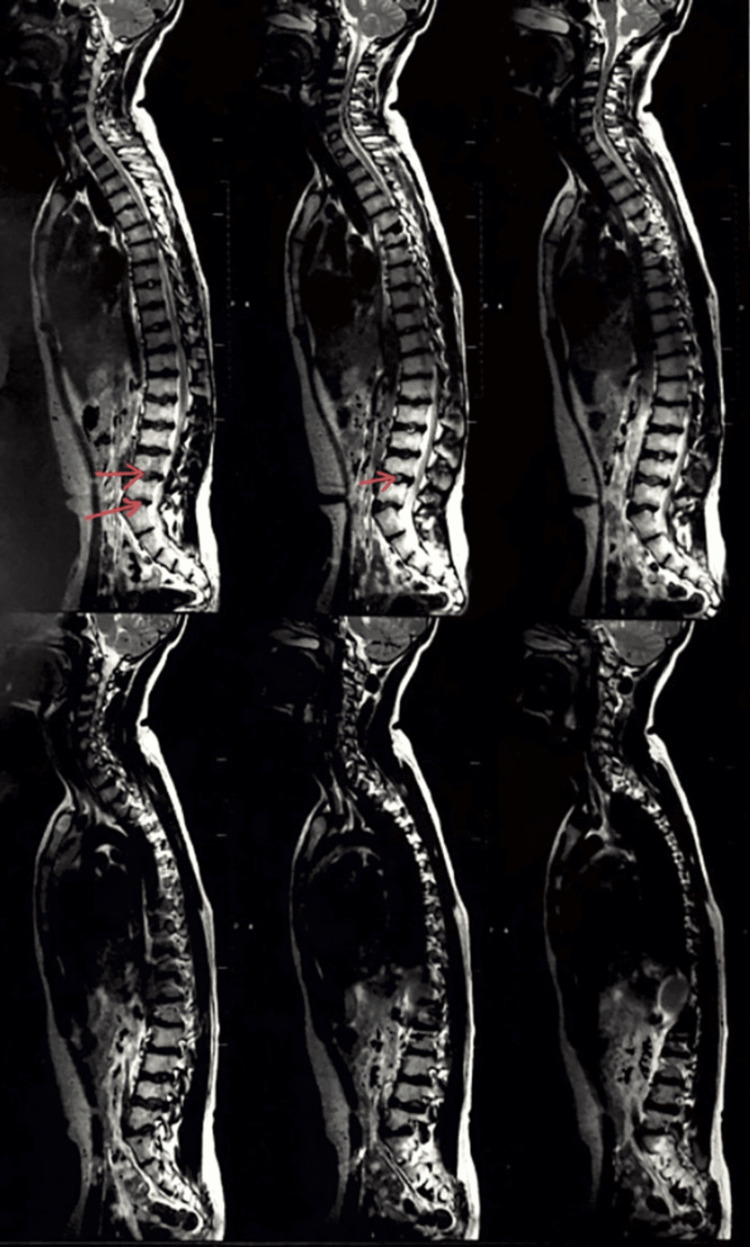
MRI showing the shmorl's nodules between the vertebral discs These red arrows show the schmorl''s nodules between the vertebral discs Image Credit: Author: Arpit Jain

X-rays and MRIs were done, which presented the following results: findings were consistent with Scheuermann’s disease. MRI of the whole spine was performed using a T2W sequence in the sagittal plane. Several observations were made, which highlighted that there was a straightening of the cervical spine with loss of normal lordosis. Degeneration was seen in intervertebral discs in the form of T2 hypointensity at multiple levels. Osteophyte (bone spur) disc complexes were seen at C3-C4, C4-C5, and C5-C6 levels. Reduced vertebral body height with end plate irregularities was seen at C3, C4, and C5 levels. The intervertebral disc spaces show normal height, and no evidence was seen of any cord abnormality. Intervertebral discs show degeneration in the form of T2 hypointensity at multiple levels. Figure 1 shows an X-Ray with scoliotic incurvation in the vertebra. Figure 2 shows the kyphotic deformity in the back. Deformity in the back was measured by the Cobb angle method. Figure 3 shows the schmorl's nodules between the vertebral discs. 

## Discussion

Scheuermann’s disease is a genetic disease, which is also referred to as Scheuermann’s kyphosis. It is considered one of the most common causes of back pain in teenagers. Though it is regarded as a genetic disorder, the exact etiology of Scheuermann’s disease is not known. There has also been speculation that this disease may be influenced by mechanical stress. The disease is a noncancerous condition that causes pain and swelling in the injured area. Patients with Scheuermann's usually start to experience symptoms between ages 20 and 40 years. The condition is rare in children but more common in adults than in children. Largely the etiology is unknown; however, some school of thought believes it to be hereditary. Physical therapy helps patients recover from the condition by reducing symptoms and improving mobility. Although the precise mode of transmission is still unknown, this illness has a hereditary component [1].

Clinicians recommend exercises to strengthen muscles, improve posture and reduce stress on injured areas, such as core strengthening and balance exercises. A patient may also use a walker or crutches during physical therapy to improve mobility while strengthening muscles for stability. Stretching exercises help strengthen muscles and reduce tension in the joints, while cold packs and massage relax tense muscles while promoting healing. The natural history of Scheuermann's disease is unknown, but it might be associated with an increased risk of back pain. The evolution of thoracolumbar and lumbar disease is unknown [2]. One reason why Scheuermann’s disease can lead to scoliosis is because of the damage it causes to the intervertebral disks. The way that Scheuermann’s disease damages the vertebrae can also result in spinal curvature. In some cases, this condition may lead to scoliosis in affected individuals. Scoliosis is a bending or deforming of one’s spine that occurs due to abnormal spinal growth in childhood or adolescence. Scoliosis is generally considered a benign condition and does not require treatment. However, some people experience severe pain, limited movement, or breathing difficulties when they are incorrectly aligned in their spines. In addition, other people experience moderate symptoms but do not seek treatment due to the high cost of surgery or their belief that their condition is harmless.

Treatment

Adolescents who have pain from Scheuermann's kyphosis typically benefit from physical treatment and a brief course of anti-inflammatory drugs. Stretching, behavioral changes and physiotherapy have shown positive results in early cases of Scheuermann’s disease. Kyphosis extending from 60 to 80 degrees require bracing, which is usually given for 12 to 24 months. This is most effective in patients who have an immature skeletal system. This usually slows down the spinal curve progression. Correction of the deformity may be necessary for individuals, whether adolescent or adult, with a progressive deformity, persistent discomfort, or neurologic deficiency. The operative management is usually done when kyphosis exceeds 75 degrees and causes deformity. Surgery is also suggested when there is some kind of neurologic compression, which causes difficulty in lifestyle. The majority of patients also see improved curve deformities toward normal as well as clinical relief [1].

Other cases and their diagnosis

A 16-year-old boy arrived at the Second XiangYa Hospital of Central South University complaining of lower back pain and a kyphotic deformity that had originated four years ago. He also expressed concern about his limited lumbar activity. In this case, the patient was diagnosed with Scheuermann’s disease. Clinical history, physical examination, radiographs, and laboratory tests were done for the same. Finally, orthopedic surgery was followed for the treatment. Post-surgery, radiographs of the patient were observed which showed significantly improved sagittal vertical axis (SVA) [6].

A 50-year-old man experienced persistent pain in his neck, middle back, and low back. The discomfort was severe, with a twitch and a burning sensation. He often tends to slant to one side. In this case, the patient was required to perform a specific set of exercises for one hour a day. The results were found to be positive and showed reduced pain and increased tone in back musculature [7].

In the case study of a 19-year-old male, the patient was advised to follow a strict home exercise routine. Exercises primarily focused on core, back and lower limb strengthening. Along with exercises, the patient also received prescriptions for medications. Daily doses of Nurokind-LC and twice-daily doses of Gemcal were recommended. The patient was currently not directed to undergo surgery but was instead advised to use the required caution and care to postpone or avoid surgery.

## Conclusions

This report presents an approach to the detection and treatment of Scheuermann’s disease in a 19-year-old male. The patient is now on conservative medications with continuous physiotherapy and a proper nutritional diet. As lower limb weakening is progressive, its management is being taken care of. Post-surgery may have its issues; most notable are infections, swelling, and nerve damage. This case, as of now, does not approach surgery and may be treated successfully just with the help of conservative medicine and physiotherapy.
